# Development and Characterization of Bigel Based on Chitosan/Sodium Caseinate Hydrogel and Soy Lecithin/Beeswax Oleogel as Carriers for Ferulic Acid

**DOI:** 10.1002/fsn3.72549

**Published:** 2026-11-03

**Authors:** Ghazal Kalantari, Fatemeh Valizadeh, Khadije Abdolmaleki

**Affiliations:** ^1^ Student Research Committee, Department of Food Science and Technology School of Nutrition Sciences and Food Technology, Kermanshah University of Medical Sciences Kermanshah Iran; ^2^ Research Center of Oils and Fats Kermanshah University of Medical Sciences Kermanshah Iran; ^3^ Nutrition Sciences and Food Technology Research Center, Health Institute Kermanshah University of Medical Sciences Kermanshah Iran

**Keywords:** bigel, ferulic acid, hydrogel, oleogel, release

## Abstract

Bigels (BGs) composed of chitosan (CS)/sodium caseinate (Na‐Cas) hydrogel (HG) and soy lecithin (L)/beeswax (BW) oleogel (OG) were developed as carriers for ferulic acid (FA). The effects of BW concentration and CS: Na‐Cas ratio on FA delivery were investigated. Increasing BW reduced swelling, with the CS‐dominant sample (BG4, 7% BW, 100:0 CS: Na‐Cas) showing the lowest swelling due to its hydrophobic network. Rheological tests suggested shear‐thinning and gel‐like behavior (*G*′ > *G*″, tanδ < 1), enhanced by higher BW and CS. Also, FTIR spectra revealed synergistic interactions between HG and OG phases, while SEM showed dense, homogeneous morphology in CS/BW‐rich BGs. In addition, release studies indicated that BW limited FA release. In the gastric phase, CS concentration correlated positively with release, whereas Na‐Cas showed a negative correlation. In contrast, in the intestinal phase, higher CS reduced release, while Na‐Cas enhanced it. These findings demonstrate BGs' structural tunability for controlled FA delivery in food matrices.

## Graphical abstract

A ferulic acid‐loaded bigel was developed by combining a chitosan/sodium caseinate hydrogel with a soy lecithin/beeswax oleogel. The bigel was evaluated under simulated gastric (pH 2) and intestinal (pH 6.8) conditions to investigate its potential for protecting ferulic acid and controlling its release. The results highlight the potential of this food‐grade bigel system as a carrier for ferulic acid delivery.

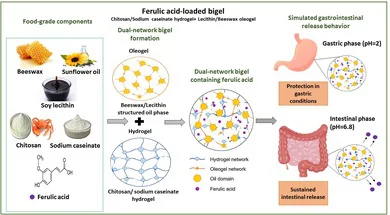

## 1 Introduction

It is undeniable that today's consumers are increasingly preferring foods with little or no artificial ingredients, driving the demand for natural products. The demand and trend are towards development of food products with bioactive compounds which not only provide health benefits but also enhance the acceptability of the food to the consumers (Vignesh et al. 2024). Food manufacturers use these compounds for their different health benefits such as antioxidant, antimicrobial, anticancer, flavor enhancing, coloring, and nutritional properties (Rezagholizade‐Shirvan et al. 2024). But there are difficulties related to poor solubility, stability, and bioavailability of these compounds that make it hard to incorporate them into food products (Fatima et al. 2024). Ferulic acid (FA) is a hydroxy derivative commonly present in vegetables and a phytochemical compound in plants. This compound has medicinal properties such as antioxidant and anti‐inflammatory effects. It plays a role in immune system regulation, cardiovascular system protection, and diabetes prevention. Despite its numerous health benefits, incorporating FA into food systems requires an efficient delivery vehicle to ensure controlled release while maintaining its structural integrity (Zheng et al. 2024).

BGs in the more recent protection procedures of different compounds are increasing. These are gels with hydrophilic three‐dimensional system matrices incorporated in the pharmaceutical sector that offer good absorbability and sustained release. These gels also provide excellent contact time and area, which are made of HG and OG. The liquid phase of HG is water, while that of OG is an organic solvent. One of the most appealing features of BGs is that they can incorporate both lipophilic and hydrophilic compounds, which may be very difficult to achieve via other techniques (Kasparaviciene et al. 2024). HGs are prepared mainly from a hydrophilic polymer network that can hold a substantial amount of liquid. This ability is determined by the presence of some functional groups that help in the diffusion of the liquid and important solutes. Due to their hydrophilicity, flexibility, elasticity, and high swellability, HGs have found applications in many fields (Hamed et al. 2023).

In this study, the hydrogelators used were chitosan (CS) and sodium caseinate (Na‐Cas). CS is a linear polysaccharide that is obtained from chitin by deacetylation. It is biocompatible, biodegradable and has antimicrobial properties which are useful in drug delivery. In this system, CS is a natural carbohydrate polymer that is crucial in regulating the stability of the gel network, modulating the release of FA and protecting the bioactive (Alimi et al. 2023). Na‐Cas is a water‐soluble protein derived from casein in milk through alkaline treatment. Its high nutritional value and binding properties also make it useful in protein supplements and biodegradable packaging materials. The combination of CS and Na‐Cas allows simultaneous exploitation of polysaccharide‐driven network stability and protein‐mediated interfacial functionality, resulting in a HG phase with improved structural adaptability for BG formation (Khan et al. 2024).

OGs consist of a gel‐forming polymer and an organic solvent to exhibit semisolid characteristics. One of the advantages of OGs is their effective ability to encapsulate lipophilic bioactive compounds with great potential in both medicine and nutrition, as healthy alternatives to solid fats (Liu et al. 2024). The oleogelators utilized in this research were lecithin (L) and beeswax (BW). L is a diglyceride (diglyceride of stearic acid, palmitic acid, and oleic acid) bound to phosphoric acid and it is present in and/or isolated from egg yolk and soybeans. In targeted delivery, recent studies have also shown the potential of L as a bioactive and biotherapeutic agent (Thy et al. 2025). The chemical composition of BW is not precisely known, but is usually accepted to be a mixture of fatty acids, polyols, and hydrocarbons that give the BW properties such as water resistance (Zhou et al. 2024).

BGs have caught the attention as delivery systems for protecting bioactive compounds from oxidation and degradation and for controlled release (Dangi et al. 2025). BGs have been utilized for probiotic bacteria, beta‐carotene, and bioactive, lipid‐soluble compounds with high levels of essential fatty acids, sterols, and fat‐soluble vitamins. The unique design of BGs makes them an ideal candidate for use in nutraceutical formulations where bioactive compounds need to be protected and released in a controlled manner through gastrointestinal phases. However, while their potential is being recognized, there is a lack of comprehensive investigations addressing the microstructural properties of these two‐component gels (Zampouni et al. 2024). Nevertheless, most reported food‐grade BG systems have mainly focused on improving physical stability or delivering specific bioactive compounds, while the relationships among formulation composition, molecular interactions, microstructure development, and functional performance remain insufficiently understood. Particularly, the combined contribution of polysaccharide–protein composite HGs and BW‐based OGs in constructing a tunable dual‐network structure has not been fully explored. CS can provide mechanical reinforcement and bioactive protection, whereas Na‐Cas contributes protein‐mediated interactions and structural flexibility; however, the optimization of their ratio within a BG system requires further investigation (Li et al. 2024; Yang, Zheng, et al. 2024). In addition, although BW is an effective oleogelator capable of forming crystalline hydrophobic domains, its role in regulating network compactness, swelling behavior, and bioactive release in combination with a hydrogel phase remains unclear. Owing to the complexity of the task, only some research has focused on the gel polymer constituents in recent years (Karmakar et al. 2023). To the best of our knowledge, this is the first report of a BG system integrating L/BW OG with CS/Na‐Cas HG for targeted FA delivery, representing a dual‐network structure that has not been previously explored in food‐grade delivery systems. The dual‐network structure of the developed BGs is formed through the synergistic combination of a hydrogen‐bonded CS/Na‐Cas HG network and a crystalline L/BW OG phase, resulting in a physically interpenetrated matrix with enhanced structural integrity and stability. Compared to earlier systems, this formulation may offer greater tunability through biopolymer composition and BW content, enabling precise control over gel strength, swelling capacity, and pH‐responsive release behavior. In the formulation variables, the concentration of BW is the most important factor that affects the crystallinity of OG and the continuity of the hydrophobic domain and thus the compactness and diffusion resistance of BG. The dual‐network matrix is able to provide mechanical stability and selective permeability ideal for gastric‐to‐intestinal delivery transitions, which are not available in conventional emulsions or single‐phase gels. Furthermore, the use of food‐grade ingredients which are GRAS‐listed is promising in the development of nutraceutical and functional food, as the existing literature is based predominantly on pharmaceutical‐grade or synthetic ingredients (Song et al. 2026). Furthermore, the use of food grade and GRAS listed ingredients opens up the possibility of using this approach in nutraceutical and functional food development, which is not the case for much of the literature available today, which mostly is directed towards pharmaceutical grade or synthetic ingredients.

## 2 Materials and Methods

### 2.1 Materials

Sunflower oil, soy lecithin, and beeswax were purchased from a local market in Kermanshah, Iran, and used without further purification. Sodium caseinate (reagent grade), chitosan (medium molecular weight, 190–310 kDa, deacetylation degree ~85%, reagent grade), and ferulic acid (≥ 99% purity, analytical grade) were obtained from Sigma‐Aldrich Chemical Co.

### 2.2 Preparation of Oleogel (OG)

OGs were prepared by heating sunflower oil to 70°C under constant stirring at 500 rpm using a magnetic stirrer. BW was added at concentrations of 3%, 5% and 7% (w/w) and stirred for 15 min until completely dissolved. Subsequently, 3% (w/w) of L was added, and stirring continued for 10 min at this temperature until completely dispersed. Finally, after cooling the mixture to 40°C, FA was then added at a concentration of 0.05% (w/w), and stirring continued for another 5 min until a uniform mixture was formed. The mixture was then allowed to reach room temperature (Agarwal et al. 2022).

### 2.3 Preparation of Hydrogel (HG)

To prepare the CS‐based HG precursor, Sodium acetate trihydrate (4 g) was gradually dissolved in 50 mL of distilled water under continuous stirring (600 rpm, 25°C). CS powder (2 g) was then slowly added to the sodium acetate solution. At this stage, the CS did not dissolve completely and a homogeneous suspension was formed. Glacial acetic acid was subsequently added dropwise under continuous stirring until the pH reached 5.5. Under these mildly acidic conditions, CS became protonated and dissolved completely, resulting in a homogeneous HG precursor. The relatively high sodium acetate concentration was selected based on preliminary optimization experiments. For the Na‐Cas solution, Na‐Cas (3 g) was dissolved in 75 mL distilled water under stirring 600 rpm for 20 min. The pH was adjusted to 5.5 using citric acid and the final volume was adjusted to 100 mL. The selected pH was identified as a compromise condition that ensures CS dispersion while minimizing Na‐Cas aggregation, thereby enabling composite HG formation with a uniform structure. The initial solutions of both precursors were adjusted to pH 5.5 and mixed in an optimized ratio (1:1). The CS‐based precursor and Na‐Cas solutions were mixed at different ratios: (100:0, 75:25, 50:50, 25:75, and 0:100) and stirred at 500 rpm for 30 min to prepare 40 mL composite HGs (Akhtar et al. 2022; Parker et al. 2015).

### 2.4 Preparation of Bigel (BG)

Each HG formulation was mixed with its corresponding OG at a 1:1 ratio. The mixture was homogenized using a high‐speed homogenizer at 14,000 rpm for 3 min at room temperature and was controlled to form the BGs (Akhtar et al. 2022). A total of seven samples were prepared: five contained 7% BW (BG1; 25:75 CS: Na‐Cas, BG2; 50:50 CS: Na‐Cas, BG3; 75:25 CS: Na‐Cas, BG4; 100:0 CS: Na‐Cas, and BG5; 0:100 CS: Na‐Cas), one contained 5% BW (BG6: 50:50 CS: Na‐Cas), and one contained 3% BW (BG7: 50:50 CS: Na‐Cas) and no phase separation was observed after homogenization. For samples with lower BW content fixed HG: OG ratio (1:1) was selected to ensure structural integrity while enabling effective interaction between hydrophilic and lipophilic domains, as commonly adopted in food‐grade bigel systems. The experimental design was structured to systematically investigate the dominant effects of BW concentration and CS: Na‐Cas ratio on BG properties while maintaining a manageable experimental matrix. The BW concentration of 7% was selected as the primary formulation for compositional screening due to its ability to form a stable OG network. Lower BW concentrations (3% and 5%) were included only at a fixed CS: Na‐Cas ratio to evaluate the isolated effect of OG strength on BG behavior. Expanding all CS: Na‐Cas ratios across multiple BW concentrations was not feasible due to material and experimental constraints.

### 2.5 pH Determination

The pH of each BG was measured by dispersing a known amount in 10 mL of distilled water. A digital pH meter (at 25°C) was used, and each measurement was repeated in triplicate to ensure the accuracy and reproducibility of the results (Sotirova et al. 2023).

### 2.6 Swelling Ratio Measurement

Approximately 5 g of the fresh BG sample was immersed in 50 mL of deionized water at 25°C for 10 h in Petri dishes. Excess water on the surface was removed using lint‐free tissue and a pipette to avoid mechanical deformation. All experiments were performed in triplicate (Yang, Song, et al. 2024). The Swelling ratio was calculated using the following equation:

$$\text{Swelling ratio}=\left(m_{1}-m_{0}\right)/{m_{0}}^{*}100$$

where *m*_0_ is the initial weight of BG, and *m*_1_ is the final weight of BG.

### 2.7 Rheological Measurement

Rheological measurements were conducted using a Haake Mars 60 rheometer (Thermo Fisher Scientific, Germany) equipped with a parallel plate geometry (35 mm diameter, 1 mm gap). A strain sweep test was performed at a fixed frequency of 1 Hz to identify the linear viscoelastic region (LVR), with strain applied in the range of 0.01% to 10%. Approximately 2.0 g of the BG sample was placed on the plate for subsequent analysis. Based on the results, the parameter strain = 0.05% was chosen as the optimal value for conducting the frequency test. Frequency sweep tests (0.01–100 Hz at a fixed strain of 0.05%) were conducted to evaluate the storage modulus (*G*') and loss modulus (*G*“) of the BGs. All measurements were performed at 25°C, and silicone oil was applied to prevent water evaporation (Kaimal and Singhal 2023).

The data were fitted to the Bohlin model (*G*' = *a*(*ω*)^*b*^) (*a*: (intercept), the structure strength, *b*: (slope), the frequency dependency index, and *ω*: angular frequency). Viscosity values reported in Table 1 were obtained from flow measurements at a shear rate of 53 s^−1^.

**TABLE 1:** pH, swelling ratio, and viscosity of BG samples.

Sample	pH	Swelling ratio%	Viscosity (Pa.s)
BG1	5.49 ± 0.082^b^	18.2 ± 0.57^b^	4984.35 ± 14.6^d^
BG2	5.42 ± 0.014^e^	14.65 ± 2.19^c^	5672.99 ± 17.4^c^
BG3	5.45 ± 0.016^c^	13.45 ± 1.91^c^	5727.59 ± 9.2^b^
BG4	5.41 ± 0.081^f^	10.8 ± 2.55^d^	5841.62 ± 15.1^a^
BG5	5.98 ± 0.102^a^	18.35 ± 1.48^b^	4938.13 ± 7.3^e^
BG6	5.49 ± 0.054^b^	18.4 ± 0.42^b^	4860.47 ± 21.5^f^
BG7	5.44 ± 0.033^d^	22.3 ± 2.69^a^	4321.42 ± 13.8^g^

*Note:* 1‐ Data are mean ± standard deviation in triplicate. Values with a similar lowercase letter are not significantly different (*p* < 0.05).

### 2.8 ATR‐FTIR Spectra

Fourier transform infrared (FTIR) spectroscopy was conducted using Irprestige, a Shimadzu FTIR spectrophotometer in attenuated total reflectance (ATR) mode, following the Zhu et al. (2021). The purpose was to identify functional groups and analyze potential physical or chemical interactions within the two‐component gel. Spectra were recorded in the range of 4000 to 400 cm^−1^, with a resolution of 4 cm^−1^, and an average of 32 scans. The instrument was interfaced with specialized software to process the spectra, which involved subtracting background signals and generating a new reference spectrum after every three scans. All measurements were performed under controlled conditions at 25°C ± 2°C (Liu et al. 2025).

### 2.9 Microstructure Observations by Scanning Electron Microscopy

The network structure of the BGs was analyzed using a (Quanta450 scanning, USA) electron microscope (SEM). For sample preparation, the BGs were affixed onto double‐sided adhesive carbon tape (NEM tape, Nisshin, Japan) and coated with a thin layer of gold/palladium using a sputter coater (Polaron, Bad Schwalbach, Germany) to enhance conductivity. The SEM was operated in high‐vacuum mode, with the spot size set to 4.0 and the accelerating voltage adjusted within the range of 5.00 kV. Images were captured at a magnification of 400×. Other imaging parameters, such as sputtering time, coating thickness, and section thickness, were kept consistent across all samples but were not individually recorded. Gold sputtering was applied to minimize charging effects and enhance image resolution without altering the intrinsic gel microstructure. (Liu et al. 2025).

### 2.10 Release

The digestion profiles of BGs in simulated gastric fluid (SGF) and simulated intestinal fluid (SIF) were analyzed to assess the release percentages of FA from the BG samples. The compositions of SGF and SIF were adapted from well‐established in vitro digestion models commonly used for evaluating the release behavior of bioactive compounds from food‐grade delivery systems. The selected enzyme concentrations were intended to simulate physiological digestive conditions rather than replicate in vivo kinetics. SGF was prepared by combining 2.0 mg/mL NaCl and 3.2 mg/mL pepsin, with the pH adjusted to 2.0 using a 0.1 M HCl solution. BGs containing FA were sectioned into small pieces (1 mm × 1 mm) using a scalpel. A 5 g portion of the sample was mixed with 45 mL of SGF and incubated at 37°C in a thermostatic shaker set at 100 rpm for 2 h to simulate gastric digestion. During this process, 5 mL of samples were collected every 30 min (at 30, 60, 90, and 120 min) to determine the FA content. After completion of gastric digestion, the remaining BG samples were mixed with 45 mL SIF, which consisted of 8.8 mg/mL NaCl, 6.8 mg/mL KH_2_PO_4_, 2.0 mg/mL pancreatin, 12 mg/mL bile salts, and 3.2 mg/mL pancreatic lipase, adjusted to pH 6.8. These samples were incubated again at 37°C in the thermostatic shaker at 100 rpm for 2 h. Similarly, 5 mL of samples were collected every 30 min (at 150, 180, 210, and 240 min) during this phase for FA measurement. After completion of the gastric and intestinal digestion processes, the digested samples were centrifuged at 8000 rpm for 10 min at 4°C to separate the supernatant, referred to as the micelle fraction. A 0.5 mL aliquot of this micelle fraction was mixed with 2 mL hexane and 1 mL ethanol, vortexed, and centrifuged again at 8000 rpm for 5 min at 4°C. The quantification of FA content followed the protocol outlined by Zhu et al. (2021). The supernatant was analyzed using a UV–Vis spectrophotometer (JENWAY‐6715, Stone, Staffordshire, United Kingdom) at a wavelength of 320 nm (Kaimal and Singhal 2023). Ferulic acid quantification was performed using a calibration curve covering the relevant concentration range, and the *R* ^2^ value of the calibration curve was 0.99. The release percentage of FA was calculated using the following equation:

$$\text{Release ratio}=C_{\text{supernatant}}/{C_{0}}^{*}100$$

where *C*_Supernatant_ is the content of FA supernatant and *C*_0_ is the initial content of FA in the BGs. This in vitro digestion model was employed to enable comparative evaluation of FA release among different BG formulations, rather than to provide an absolute prediction of in vivo bioavailability.

### 2.11 In Vitro Release Kinetic Modeling of FA From BG Systems

To further elucidate the release mechanism of FA from the developed BG systems, the release data obtained during the simulated gastric fluid (SGF) and simulated intestinal fluid (SIF) phases were fitted to zero‐order, first‐order, Higuchi, and Korsmeyer–Peppas kinetic models. These kinetic models are widely used to describe the release behavior of bioactive compounds from delivery systems, particularly when the release mechanism is complex or involves multiple transport processes.

Since the release conditions differed between the SGF and SIF phases, the release kinetics were analyzed independently for each phase rather than using the cumulative release profile throughout the entire gastrointestinal simulation. Therefore, the release data of the intestinal phase were fitted from the beginning of the SIF stage (0% release). The model with the highest coefficient of determination (*R* ^2^) was considered the most appropriate model for describing the release behavior of FA from the BG formulations.

The zero‐order model describes a constant release rate independent of the concentration of the released compound, whereas the first‐order model assumes that the release rate is concentration‐dependent. The Higuchi model is commonly used to evaluate diffusion‐controlled release from matrix systems. The Korsmeyer–Peppas model was used to identify the dominant release mechanism based on the release exponent (n), which can indicate Fickian diffusion, anomalous transport, or Case II transport. The mathematical equations of the kinetic models are presented below:

(1) Zero–order model:Mt/M∞=kt

(2)

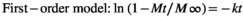

(3) $$\text{Higuchi model}:\mathrm{Mt}/M\infty ={\mathrm{kt}}^{1/2}$$

(4) $$\text{Korsmeyer}–\text{Peppas model}:\mathrm{Mt}/M\infty ={\mathrm{kt}}^{n}$$

where *Mt* represents the amount of ferulic acid released at *t*, *M ∞* denotes the total amount of ferulic acid released at infinite time, *k* is the release rate constant, and *n* is the diffusional exponent (Wang et al. 2026).

### 2.12 Statistical Analysis

The statistical significance of the study results was analyzed by using one‐way analysis of variance (ANOVA) using SPSS 20 software. A multiple comparison test was used to compare different formulations, and 95% confidence intervals, *p*‐values < 0.05, were considered to be significant.

## 3 Results and Discussion

### 3.1 pH Analysis

Among all the samples, the lowest pH (5.41) was observed in sample BG4, while the highest pH (5.98) was found in sample BG5. The pH values of the BG samples ranged between 5.41 and 5.98 (Table 1), indicating a slightly acidic environment. Among the formulations, BG5 exhibited a relatively higher pH value compared with other samples, which may be associated with its HG composition containing Na‐Cas as the sole biopolymer component. Compared with CS, Na‐Cas has a different distribution of ionizable functional groups and buffering behavior, which can influence the final pH of the system after acid adjustment. The absence of CS, which contains protonatable amino groups, may reduce the contribution of amino group protonation to the acidic balance of the HG phase. Therefore, differences in CS/Na‐Cas ratios may contribute to the observed variation in pH among BG formulations (Zhao et al. 2026). This was likely attributed to the use of citric and acetic acids in the preparation of the HG. Although this range did not match the highly acidic pH of the gastric phase (~2), it remained within the physiologically acceptable range for oral delivery systems. Such mildly acidic environments have been suggested to be beneficial for mucosal compatibility, as extremely low pH can induce tissue irritation and reduce the stability of biopolymeric structures. Furthermore, keeping this range of pH can help to minimize the degradation of the gel matrix before it is supposed to. It can, therefore, help to control the release of FA, particularly in the intestinal phase, when pH increases. The stability of the sensitive bioactives such as FA may depend on the balanced pH in this range as it prevents acid‐catalyzed degradation and helps to maintain the gel network integrity during gastrointestinal transit (Sotirova et al. 2023).

### 3.2 Swelling Ratio

The swelling behavior of the BGs varied depending on the formulation (Table 1). The lowest percentage of BG4 was 10.8%, while the highest one for BG7 was 22.3%. Thus, the sample with the HG part of 7% of BW and 100% of CS demonstrated the lowest level of swelling. BG4, containing 7% BW and 100% CS in the HG component, exhibited the lowest swelling. This may be due to its dense, hydrophobic network structure. In contrast, BG7, with the lowest BW content and a balanced CS: Na‐Cas ratio (50:50), demonstrated the highest swelling. This could be due to its looser network and the hydrophilic nature of Na‐Cas. An apparent inverse relationship was observed between the BW concentration and swelling ratio. Higher BW concentration (e.g., in samples with 7% BW) appeared to reduce the swelling ratio, possibly due to the creation of a strong and water‐impermeable texture by BW. BW's crystalline and lipophilic structure may act as a physical barrier against water diffusion into the gel network. It might also reinforce the gel network by increasing the compactness of the matrix, restricting water penetration. Additionally, a higher BW concentration may limit water uptake, thereby reducing gastric fluid penetration into the BG matrix.

This swelling behavior was closely related to the structural characteristics of the BG network. Formulations containing higher BW and CS levels generally exhibited more compact structures, which were consistent with their higher viscosity and elastic behavior observed in rheological analysis. These structural features may originate from stronger intermolecular interactions among HG and OG components, as suggested by FTIR results, limiting network expansion and water diffusion. Therefore, reduced swelling in compact BG networks may contribute to slower FA migration and more controlled release behavior (Oyom et al. 2025; Hernández‐Nolasco et al. 2025).

Zheng et al. (2020) reported that high BW concentration in the BG structure prevented water penetration and reduced swelling ratio, while lower BW concentrations resulted in significantly higher swelling ratios (Zheng et al. 2020). Similarly, Li et al. (2023) found that high concentrations of oleogelators (such as BW) reduced the swelling ratio (Li et al. 2023).

In samples with the same BW concentration, increasing the proportion of Na‐Cas and decreasing CS was generally associated with a higher swelling ratio. This may be due to the Na‐Cas's solubility in water compared to CS's insolubility. Cazorla‐Luna et al. (2025) reported that CS‐based BG systems absorbed only small amounts of water and exhibited limited swelling due to their inherent water‐insolubility (Cazorla‐Luna et al. 2025).

Limited swelling of CS may also be linked to its semicrystalline structure, which restricts free volume within the polymer matrix for water uptake. Conversely, Na‐Cas, as a water‐soluble protein, may enhance swelling due to its hydrophilic and disordered structure. Walendziak et al. (2025) found that the swelling ratio of BGs depended significantly on polymer concentration (Walendziak et al. 2025). Na‐Cas gels, when submerged in water, absorbed large amounts of water due to network entanglement, potentially increasing osmotic swelling pressure and accelerating release rate in aqueous environments (Kaimal and Singhal 2023).

### 3.3 Rheological Properties

All BG samples demonstrated non‐Newtonian, shear–thinning (pseudoplastic) behavior, as viscosity decreased with increasing shear rate (Figure 1). This behavior results from partial alignment and disruption of weak internal bonds under shear stress, facilitating flow which is typical of structured soft materials. Hamed et al. (2023) also suggested the pseudoplastic (shear‐thinning) behavior of the BGs. Such rheological behavior is advantageous for delivery applications, as it enables ease of processing during manufacturing while maintaining structural stability during storage and administration (Hamed et al. 2023).

**FIGURE 1:**
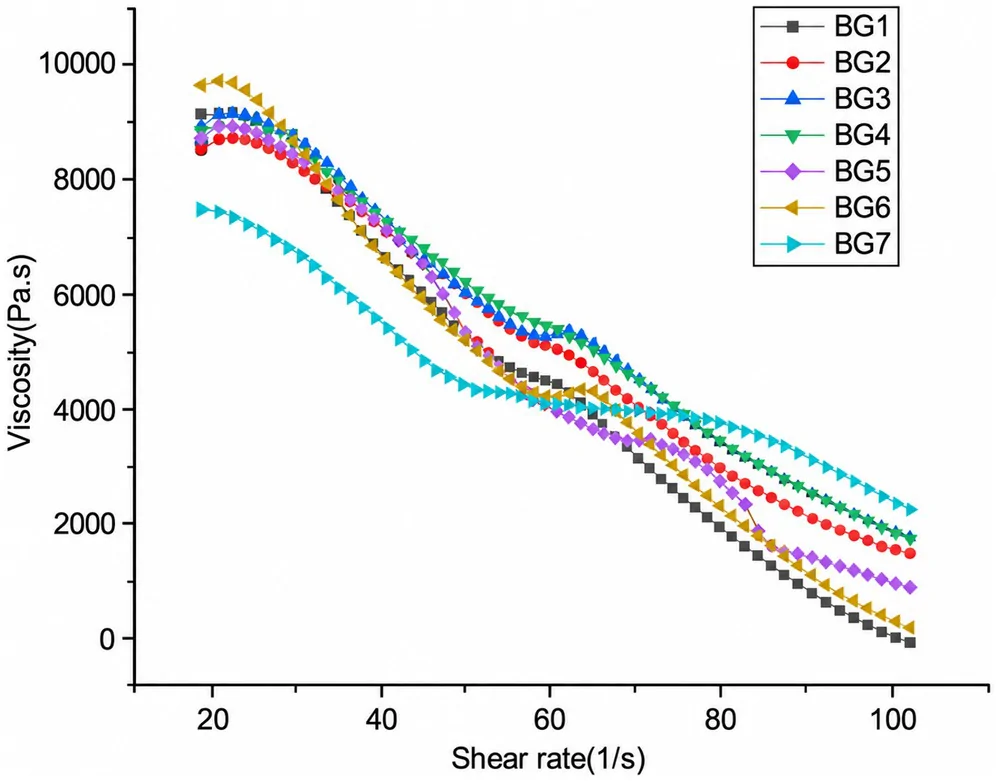
The viscosity chart of BG samples.

Sample BG4 exhibited the highest viscosity at a shear rate of 53 s^−1^ (Table 1), likely due to stronger crystalline and electrostatic interactions, indicating a dense, well‐structured internal network. In contrast, BG7 showed the lowest viscosity, suggesting weaker network formation and reduced structural integrity. The lower viscosity and weaker viscoelastic response of BG7 may be related to its formulation characteristics, including the lower BW content and the presence of a higher Na‐Cas proportion. The reduced BW concentration may decrease the contribution of crystalline lipid reinforcement to the BG network, while the increased Na‐Cas content may provide a more flexible but less rigid protein‐based structure compared with CS. This interpretation was consistent with the higher swelling ratio and heterogeneous morphology observed for BG7, suggesting a less compact network with greater accessibility of aqueous media (Qiu et al. 2024; Farooq and Ahmad 2022). These differences might be linked to compositional variation, particularly BW and CS content. Consistent with these observations increasing BW concentration has been reported to enhance viscosity and network strength in CS/Na‐Cas emulsion films, likely due to the formation of larger sized particles (Akhtar et al. 2022). Cazorla‐Luna et al. (2025) reported that incorporating CS into the BG improved mechanical properties through hydrogen bonding and electrostatic interactions, reinforcing the gel matrix (Cazorla‐Luna et al. 2025).

Among samples containing 7% BW, BG5 had the lowest viscosity, possibly due to its inability to form a stable network, possibly due to the suboptimal CS: Na‐Cas ratio, which disrupted gel matrix cohesion. Similarly, Yang, Song, et al. (2024), Yang, Xu, et al. (2024), Yang, Zheng, et al. (2024) observed that increasing wax concentration in BGs based on pea protein HG and rice bran wax OG improved viscosity and consistency (Yang, Xu, et al. 2024). As shown in Figure 2A,B, in all samples, *G*' was greater than *G*“, indicating elastic‐dominant gel‐like behavior. Alkabaa et al. (2024) suggested that the loss factor (tanδ) provides important information about the viscoelastic behavior of the BGs, and in all samples, tanδ < 1, and it indicated dominant elastic (solid‐like) behavior. This dominant elasticity is often favorable in controlled‐release formulations where structural integrity over time is required and may help prevent fast release of encapsulated compounds during storage, which is critical for product shelf life and functional performance (Alkabaa et al. 2024).

**FIGURE 2:**
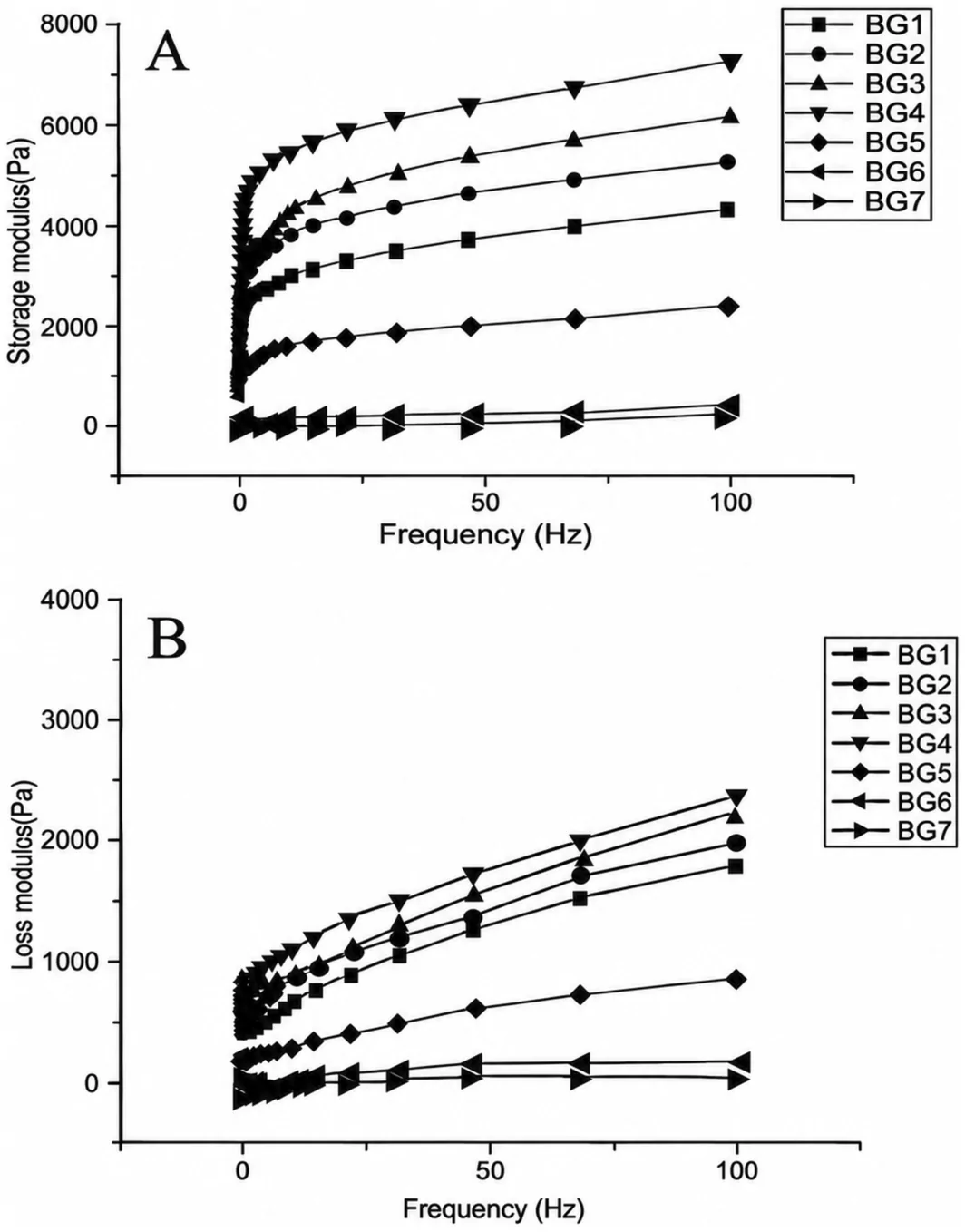
Frequency sweep test of BGs (A) storage modulus (*G*'), (B) loss modulus (*G*“).

In all samples, *G*' and *G*“ increased with frequency, showing frequency‐dependent behavior typical of structured gels. As shown in Table 2, BW concentration and the CS‐to‐Na‐Cas ratio appeared to influence the rheological properties. The power law model (*G*' = aɷ^b^) was fitted to the frequency sweep data. As shown in Table 2, a value (structure strength) tended to increase with BW content, suggesting stronger gel networks due to the crystalline structuring. BG4 had the highest a value, suggesting its robust internal matrix. Samples with higher Na‐Cas ratios (e.g., BG1 and BG5) showed the lowest structure strength due to the protein's amphiphilic and less cohesive nature, which may have disrupted the gel's internal order. Excess Na‐Cas could interface with hydrogen bonding and ionic interactions, leading to less interconnected networks. The b value (frequency dependence) was highest in BG7, reflecting a less stable and more frequency‐sensitive network. Lower b values were observed in BG4 and BG1, which suggested more frequency‐independent (stable) gels. This could mean that these gels maintained their structure under different oscillatory frequencies, which would be desirable for mechanical resilience during processing or storage.

**TABLE 2:** The power law (frequency sweep test) parameters of BG samples.

Sample	*a* (Pa × s/rad)	*b*	*R* ^2^
BG1	1936.1	0.11	0.99
BG2	2392.5	0.12	0.99
BG3	2504.5	0.13	0.99
BG4	3444.4	0.11	0.98
BG5	1066.1	0.11	0.99
BG6	118.4	0.12	0.99
BG7	4.9	0.18	0.94

These findings aligned with the notion that CS may contribute to stronger, more elastic structures through polyelectrolyte complexation, while Na‐Cas may provide flexibility but reduce structural integrity due to its disordered nature. Overall, the rheological analysis suggested that both BW content and CS: Na‐Cas ratio critically affect BG mechanical strength, stability, and viscoelastic properties of BGs. The relationship between formulation composition, intermolecular interactions, microstructure, and rheological behavior further explains the observed differences among BG formulations. The higher viscosity and structural strength observed in BW‐ and CS‐rich samples may be attributed to enhanced network compactness through crystalline reinforcement of BW and interactions among CS, Na‐Cas, and lipid components. These structural characteristics were consistent with SEM and FTIR findings, which indicated improved network organization and compatibility between HG and OG phases. Consequently, variations in network strength influenced water penetration, swelling behavior, and FA diffusion through the BG matrix (Murru et al. 2022; Li et al. 2024).

Compared with previously reported BG systems, the formulations developed in this study achieved elastic‐dominant behavior (*G*′ > *G*″) at relatively moderate BW concentrations. For instance, Yang, Song, et al. (2024), Yang, Xu, et al. (2024), Yang, Zheng, et al. (2024) reported that pea protein–based BGs required higher wax contents to reach comparable viscoelastic strength, while Kasparaviciene et al. (2024) observed reduced mechanical stability in protein‐based BGs lacking polysaccharide reinforcement (Yang, Xu, et al. 2024; Kasparaviciene et al. 2024). The present CS/Na‐Cas–BW system benefits from the synergistic combination of a polysaccharide–protein hydrogel and a crystalline wax‐based OG, resulting in a mechanically robust dual‐network structure suitable for controlled delivery applications.

Rheological measurements were performed at a single temperature (25°C), to allow comparison under ambient conditions. Temperature‐dependent behavior was not evaluated and may provide additional insight into the thermal stability of the BG network during food processing and storage. This aspect is suggested for future investigations.

### 3.4 ATR‐FTIR Analysis

ATR‐FTIR spectroscopy was employed to investigate intermolecular interactions between the HG and OG phases in the BGs. The recorded spectra (Figure 3) revealed characteristic bands originating from CS, Na‐Cas, L, BW, and FA.

**FIGURE 3:**
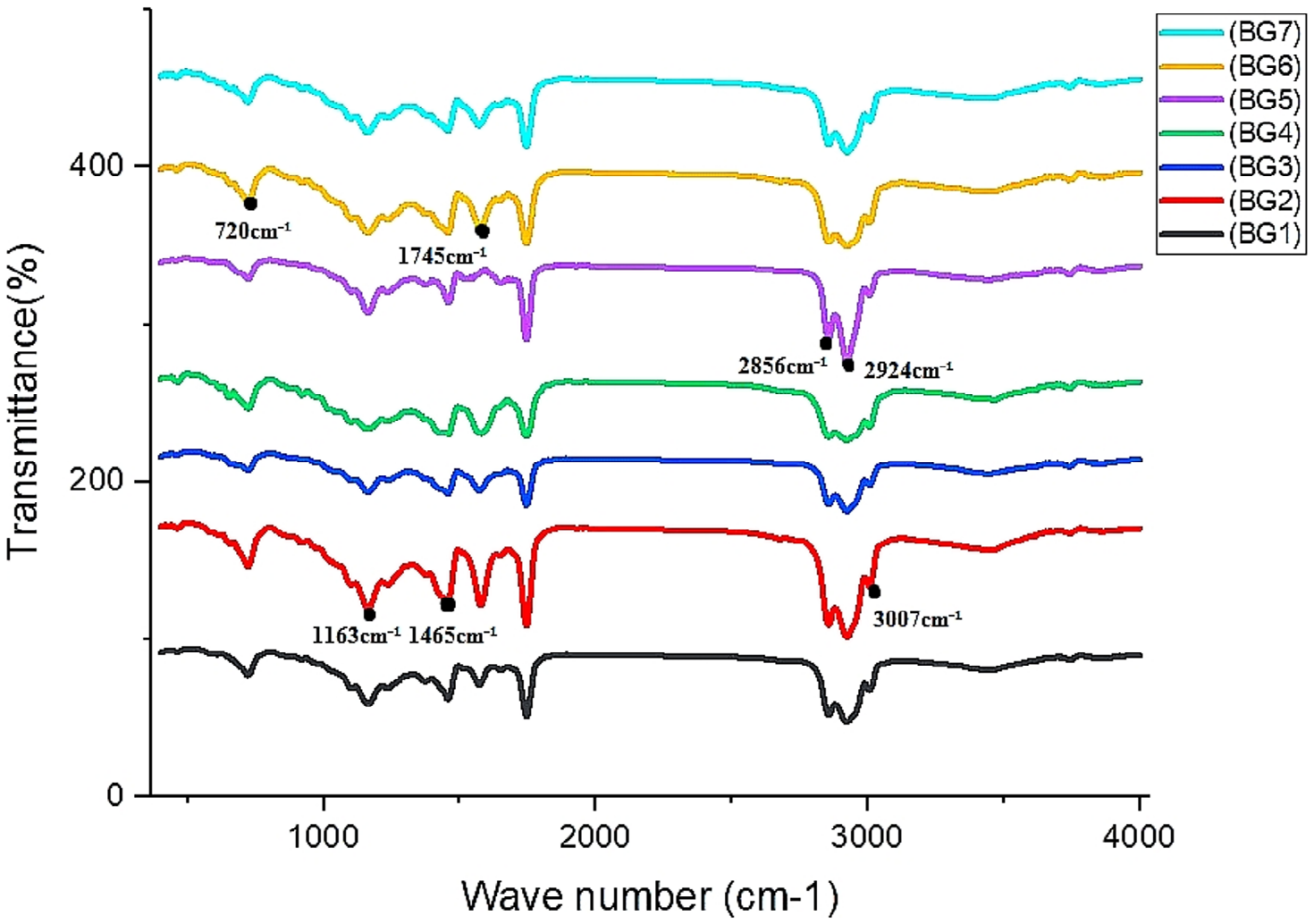
FTIR‐ATR spectra of BGs.

Rather than indicating new covalent bond formation, the observed peak overlaps, shifts, and intensity changes suggest the presence of secondary interactions, including hydrogen bonding, electrostatic attractions, and hydrophobic associations among HG and OG components. These interactions are critical for stabilizing the physically interpenetrated dual‐network structure of the BGs. The incorporation of BW as a gelling agent introduced a peak at 720 cm^−1^, which may arise from the amide groups in BW and bending vibrations of the CH_2_ groups in the hydrocarbon chains present in both BW and L. According to Zhou et al. (2024), the addition of BW and the presence of amide groups in its structure could contribute to the formation of this peak. This may indicate enhanced structural integration of the BW within the lipid network, forming secondary interactions that reinforce the gel framework (Zhou et al. 2024). A peak was identified at 1465 cm^−1^, as reported by Loza‐Rodríguez et al. (2023), which was commonly associated with the CH2 and CH3 bending in the aliphatic chains of fatty acids, suggesting the presence of both saturated and unsaturated lipids from both BW and sunflower oil (Loza‐Rodríguez et al. 2023; Liu et al. 2025).

Peaks at 1163 and 1745 cm^−1^ were associated with C‐O and C=O stretching vibrations, characteristic of ester, alcohol, and ether groups. These peaks likely originated from phospholipids in L and BW, while contributions from CS and Na‐Cas may include hydroxyl, amide, and carboxyl groups. Shaikh et al. (2022) explained that the same peaks might result from the interaction of C=O stretching in sunflower oil, lipids, and fatty acids. The observed absorption bands might therefore originate from various functional groups, which might indicate their compatibility in the binary mixture, as well as promoting the homogeneity of both hydrophilic and lipophilic domains in the matrix (Shaikh et al. 2022; Zheng et al. 2020). CS may influence the formation of hydroxyl and ether groups, whereas Na‐Cas can contribute through its amide and hydroxyl groups. Interactions among these components may enhance observed vibrations (Liu et al. 2025).

L and BW, as well as CS and Na‐Cas, could form weak bonds and hydrogen interactions with each other, intensifying stretching vibrations. FA may also enhance these peaks due to its carbonyl groups (Akhtar et al. 2022). This indicated a synergistic effect of these compounds in the spectral characteristics at 2856 and 2924 cm^−1^ in all BG samples. Such synergy may form a more cohesive, homogenously distributed gel matrix, essential for phase stability and mechanical integrity. Liu et al. (2025) attributed these peaks to stretching vibrations of C–H and C=H bonds in the acyl chains of saturated and unsaturated fatty acids (Liu et al. 2025; Shaikh et al. 2022).

The peak at 2856 cm^−1^ corresponded to symmetric CH_2_ stretching in hydrocarbon chains, influenced by L and BW, with a possible contribution from Na‐Cas and CS (Liu et al. 2025; Li et al. 2024). The peak at 2924 cm^−1^ was related to asymmetric stretching vibrations and hydrogen interactions between CH_2_ groups in L, and this peak was sharper in the sample containing only Na‐Cas in its HG, possibly highlighting its role and the interactions with L, BW, and Na‐Cas in enhancing these vibrations (Akhtar et al. 2022; Li et al. 2024). The coexistence and partial overlap of lipid‐ and polymer‐associated bands indicate strong compatibility between the OG and HG phases, supporting effective phase integration rather than phase separation within the BG matrix. A peak at 3007 cm^−1^ corresponded to CH stretching in aromatic rings, attributed to the phenolic structure of FA. Its presence may confirm successful FA incorporation and potential for hydrogen bonding with both HG and OG phases. Interactions among all components could intensify vibrational bands, indicating synergistic bonding between CS, Na‐Cas, L, and BW (Valizade et al. 2026).

Ardestani et al. (2023) suggested that this peak formation may relate to hydrogen bonding between Na‐Cas hydroxyl groups and other components. This network likely enhances the phase important for bioactive stability or controlled release by preventing phase separation and maintaining functional integrity over product shelf life (Ardestani et al. 2023). This analysis provided molecular level evidence of compatibility and interaction among the gel constituents, supporting the formation of a physically and chemically stable BG network. These molecular interactions may contribute to the formation of a more cohesive network by restricting polymer chain mobility and improving phase integration. The enhanced network cohesion can explain the higher elastic behavior observed in formulations with stronger intermolecular interactions and may subsequently affect swelling capacity and FA release by limiting water diffusion and molecular migration (Lin et al. 2025).

Such molecular insights are pivotal for rational design of biphasic gels with tailored functional properties, especially in biomedical or nutraceutical applications. The observed shifts and intensity changes in characteristic bands suggest the formation of hydrogen bonding and electrostatic interactions between FA and CS/Na‐Cas matrix, as well as physical interactions with the OG phase, providing spectroscopic evidence for the stabilization of the dual‐network structure (Zhou et al. 2024).

### 3.5 Microscopic Observation

Scanning electron microscopy (SEM) was used to evaluate the microstructural features of the BG formulation (Figure 4). Based on the SEM observations, the BG system can be classified as a HG‐continuous matrix with dispersed oleogel domains rather than a fully bicontinuous structure. The continuous polymeric network formed by CS/Na‐Cas provides the main diffusion barrier, while the embedded BW‐based OG domains act as hydrophobic reservoirs that retard FA migration. Distinct differences in surface morphology were observed depending on the CS to Na‐Cas and BW concentration. In sample BG1, some microcracked regions were observed together with a heterogeneous particle‐size distribution consisting of both coarse and fine particles. These structural features may reflect a relatively less cohesive network associated with Na‐Cas rich HG composition. Overall, the release in this sample appeared faster than in samples with more CS (among the 7% BW samples), which may be associated with the presence of coarse particles and local structural discontinuities. However, it still seemed slower than in samples with less CS or less BW (Li et al. 2024).

**FIGURE 4:**
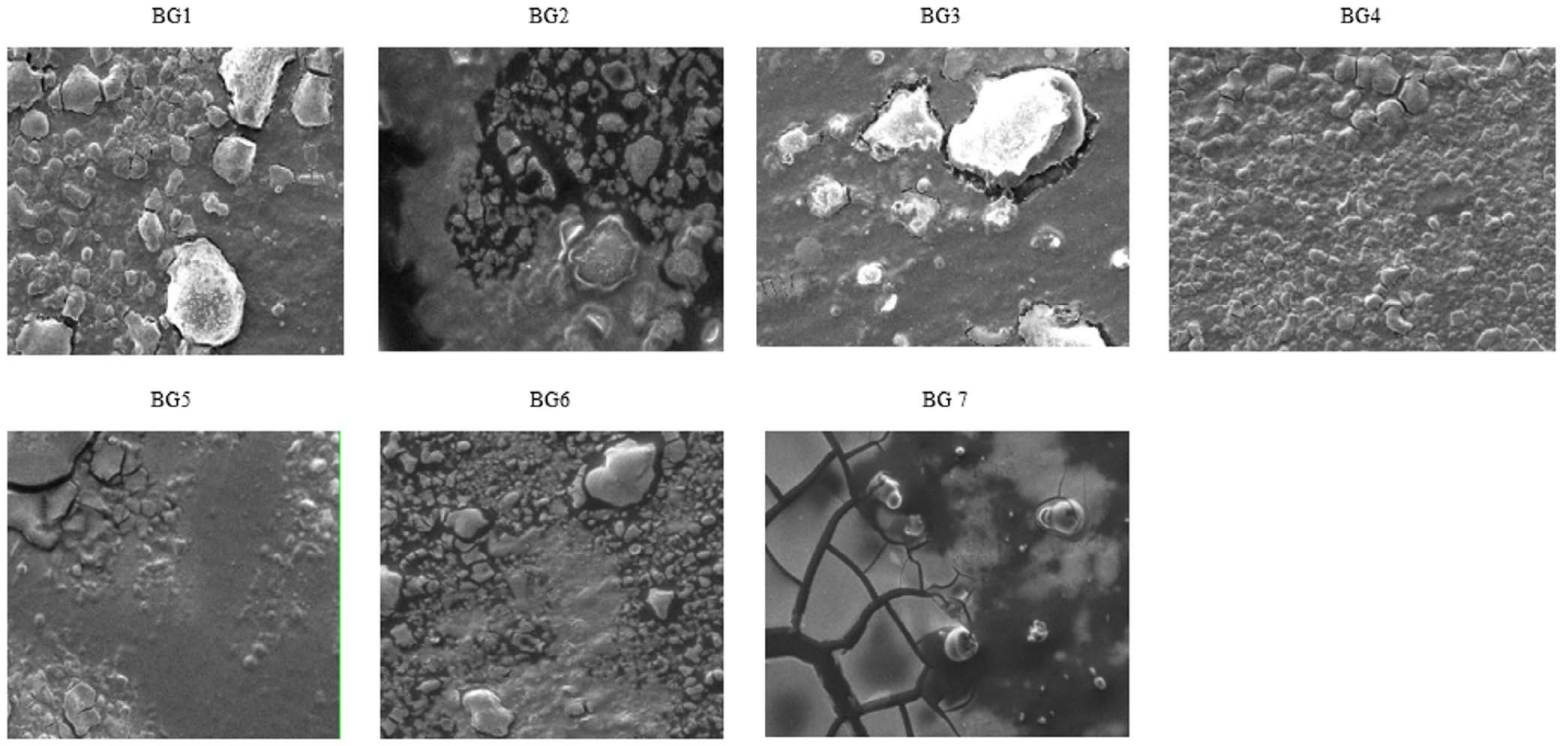
SEM pictures of BGs (scale bar: 200 μm; magnification: 400×).

In sample BG2, dense regions with fine particles, along with droplets and coarse particles, were observed. The dense regions and fine particles may indicate the formation of a relatively compact and cohesive CS‐rich network, which could contribute to more controlled FA release. Conversely, the presence of droplets and coarse particles suggests local structural heterogeneity, which may be associated with the incorporation of Na‐Cas and could facilitate FA diffusion compared with more compact regions (Xu et al. 2024).

In sample BG3, coarse particles and clumps were observed, suggesting a more heterogeneous microstructure compared with BG4. Such structural heterogeneity may have contributed to the relatively faster FA release by providing less compact regions for fluid penetration and diffusion (Li et al. 2024). Nevertheless, some compact regions were still observed, indicating partial preservation of the gel network despite the heterogeneous morphology. The relatively higher CS content compared with Na‐Cas may have contributed to maintaining network cohesion and partially controlling FA release (Li et al. 2025). However, the presence of Na‐Cas, even at a low proportion, may have introduced local structural heterogeneity resulting in a moderately higher release rate.

In sample BG4, fine and closely packed particles were observed, possibly indicating the formation of a highly compact gel network. This could be due to CS being the sole HG component and the high concentration of BW (Akhtar et al. 2022). Zhang et al. (2022) reported that iodine‐CS exhibited a smooth surface without cracks or cavities (Zhang et al. 2022). Similarly, no obvious cracks or large cavities were observed in BG4; however, the surface displayed a relatively homogeneous morphology with a rough texture. The densely packed particles may have contributed to a more controlled and slower release of FA, as molecules would need to travel a longer diffusion path through the gel network. However, Akhtar et al. (2022) reported that incorporating wax into CS/Na‐Cas emulsion films led to surface roughness and structural discontinuities due to the wax's hydrophobic nature and phase immiscibility (Akhtar et al. 2022). In another research done by Pal and Agarwal (2024), SEM images revealed that in the film based on guar gum and BW developed for cheese packaging and shelf‐life extension, the presence of BW resulted in a smooth, dense, and pore‐free surface (Pal and Agarwal 2024).

In the BG5 sample, the surface exhibited relatively smooth regions with a fairly homogeneous morphology, suggesting the formation of a compact gel network. This morphology may be attributed to the high concentration of well dispersed BW, which can contribute to the formation of a dense and stable structure (Pal and Agarwal 2024). Although the surface was generally uniform, some microcracked areas were observed, which may be associated with the high Na‐Cas content. The relatively smooth morphology may contribute to a slower FA release by providing a more compact diffusion pathway, whereas the presence of microcracked regions could facilitate the diffusion of FA molecules and enhance release rate (Xu et al. 2024). Mohamed and Ramaswamy (2024) reported that Na‐Cas alone could form a brittle network, while the incorporation of carboxymethyl chitosan reduced the formation of microcracks and pores through hydrogen bonding interactions, resulting in a smoother and more compact film structure.

In BG6, small and densely packed particles were observed throughout most of the surface, suggesting a relatively compact and stable gel network compared to BG7. However, some regions showed relatively coarser particles and surface heterogeneity, likely due to an imbalance between hydrophobic and hydrophilic interactions. Smaller particles could result in slower and more controlled FA release, while coarser particles may have led to faster release (Zhou et al. 2024).

In BG7, a heterogeneous structure with dispersed white droplets was observed, indicating a weaker and less stable gel network. The heterogeneous morphology was likely associated with an imbalance between hydrophobic and hydrophilic interactions, which may have contributed to faster FA release compared to the other samples. In conclusion, higher concentrations of BW and CS in BGs may promote the formation of more compact and homogeneous gel networks with enhanced structural stability (Zhu et al. 2021; Li et al. 2024). The compact and homogeneous microstructures observed in SEM images may be attributed to the synergistic interactions between the HG and OG phases, where the interpenetrated dual‐network restricts phase separation and enhances the structural stability of the BG matrix. In contrast, it was found that increasing the amount of Na‐Cas tends to increase the structural heterogeneity, which may be beneficial for the release of FA, especially in the intestinal tract. It is concluded that optimizing the composition ratios is essential for obtaining the required release profiles, as well as for storage stability due to reducing the risk of phase separation or aggregation. The uniformity of particle distribution may also be beneficial for obtaining consistent release properties in different batches of the obtained formulation (Walendziak et al. 2025; Akhtar et al. 2022). The observed microstructural differences were consistent with the rheological, swelling and release results, indicating that compact and homogeneous networks generally exhibited higher structural strength, lower swelling and more controlled release. Therefore, the SEM observations provide additional evidence supporting the composition‐dependent relationship between intermolecular interactions, network architecture and functional performance of the BGs (Lin et al. 2025).

### 3.6 Release

The cumulative release profiles of ferulic acid were analyzed to provide a direct measure of release performance. The cumulative release of ferulic acid from all formulations is shown in Figure 5, during the simulated phases of the study, samples containing a lower percentage of BW (BG6 and BG7) generally exhibited a higher release rate compared to samples containing 7% BW in both phases. This difference may be attributed to the formation of a stronger and denser BG network at higher BW concentrations, which increased structural resistance and reduced FA mobility within the matrix, thereby delaying its release. According to Chen et al. (2022), increasing the content of BW in Pickering emulsions allowed to improve the stability, intensify the mouthfeel, and promote the controlled release of flavor compounds (Chen et al. 2022).

**FIGURE 5:**
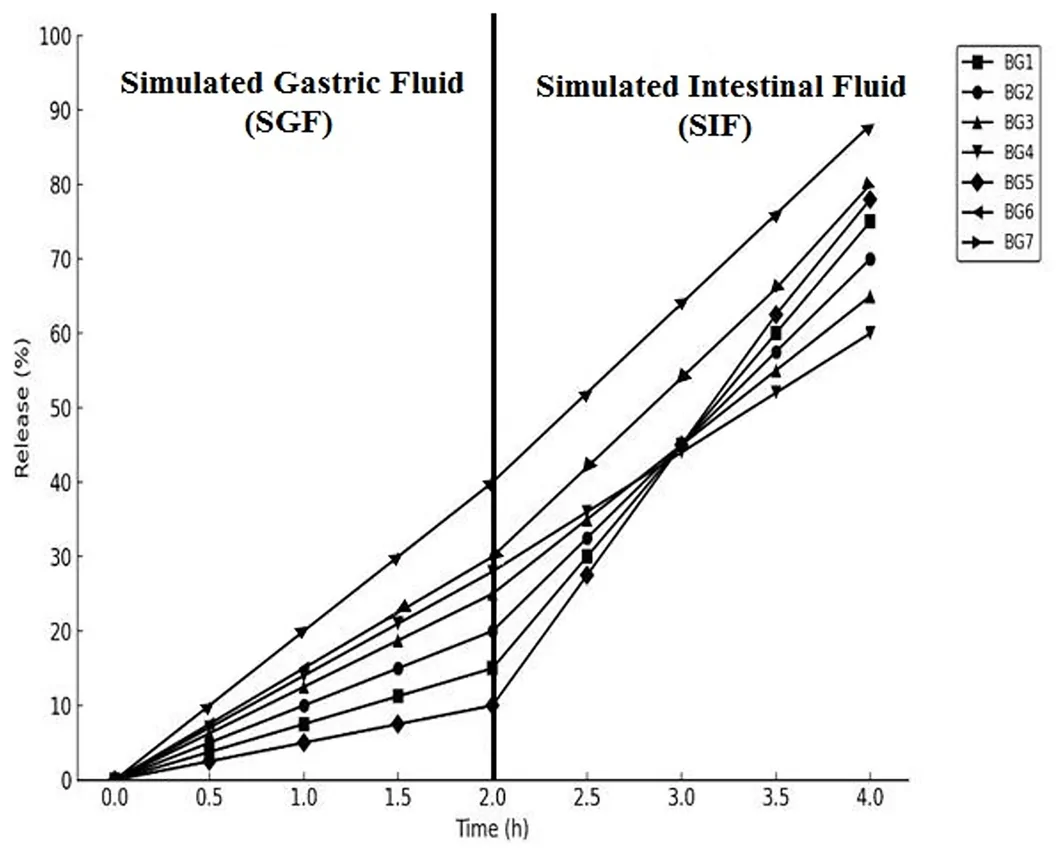
In vitro gastrointestinal release profile of ferulic acid.

To quantitatively elucidate the release mechanism of FA from the developed BG systems, kinetic modeling was performed independently for the simulated gastric and intestinal phases. The release data of each phase were fitted separately to zero‐order, first‐order, Higuchi, and Korsmeyer–Peppas models because the transition from SGF to SIF resulted in a significant change in pH and physicochemical conditions. Therefore, the intestinal phase was analyzed from its own initial release point rather than using the cumulative release profile of the entire digestion process. The calculated kinetic parameters, including *k*, *R* ^2^, and n values, are summarized in Table 3.

**TABLE 3:** Kinetic parameters of ferulic acid release from bigel samples in simulated gastrointestinal fluids.

Sample (Phase)	Zero‐order	First‐order	Higuchi	Korsmeyer‐Peppas
BG1 (SGF)	*K* = 7.5895	*K* = 0.084	*K* = 0.0618	*K* = 0.500 *n* = 0.9964
	*R* ^2^ = 1.000	*R* ^2^ = 0.9996	*R* ^2^ = 0.9903	*R* ^2^ = 1.0000
BG1 (SIF)	*K* = 29.964	*K* = 0.50	*K* = 0.0157	*K* = 0.500 *n* = 0.9978
	*R* ^2^ = 1.000	*R* ^2^ = 0.9874	*R* ^2^ = 0.9905	*R* ^2^ = 1.0000
BG2 (SGF)	*K* = 9.9881	*K* = 0.114	*K* = 0.0469	*K* = 0.504 *n* = 0.9781
	*R* ^2^ = 0.9999	*R* ^2^ = 0.9989	*R* ^2^ = 0.9985	*R* ^2^ = 0.9999
BG2 (SIF)	*K* = 24.905	*K* = 0.37	*K* = 0.0189	*K* = 0.501 *n* = 1.000
	*R* ^2^ = 0.9999	*R* ^2^ = 0.9944	*R* ^2^ = 0.9918	*R* ^2^ = 0.9998
BG3 (SGF)	*K* = 12.272	*K* = 0.145	*K* = 0.0382	*K* = 0.506 *n* = 0.9792
	*R* ^2^ = 0.9997	*R* ^2^ = 0.9986	*R* ^2^ = 0.989	*R* ^2^ = 0.9996
BG3 (SIF)	*K* = 19.952	*K* = 0.270	*K* = 0.0235	*K* = 0.505 *n* = 0.9919
	*R* ^2^ = 0.9997	*R* ^2^ = 0.9973	*R* ^2^ = 0.9926	*R* ^2^ = 0.9997
BG4 (SGF)	*K* = 13.998	*K* = 0.171	*K* = 0.0335	*K* = 0.506 *n* = 0.9687
	*R* ^2^ = 0.9998	*R* ^2^ = 0.9974	*R* ^2^ = 0.9871	*R* ^2^ = 0.9995
BG4 (SIF)	*K* = 16.064	*K* = 0.202	*K* = 0.0292	*K* = 0.506 *n* = 0.9984
	*R* ^2^ = 0.9997	*R* ^2^ = 0.9970	*R* ^2^ = 0.9876	*R* ^2^ = 0.9996
BG5 (SGF)	*K* = 5.1432	*K* = 0.054	*K* = 0.0913	*K* = 0.506 *n* = 1.0000
	*R* ^2^ = 0.9995	*R* ^2^ = 0.9997	*R* ^2^ = 0.9932	*R* ^2^ = 0.9999
BG5 (SIF)	*K* = 33.735	*K* = 0.629	*K* = 0.0139	*K* = 0.512 *n* = 0.9839
	*R* ^2^ = 0.999	*R* ^2^ = 0.9885	*R* ^2^ = 0.9946	*R* ^2^ = 0.9996
BG6 (SGF)	*K* = 15.143	*K* = 0.188	*K* = 0.031	*K* = 0.508 *n* = 0.9849
	*R* ^2^ = 0.9994	*R* ^2^ = 0.9988	*R* ^2^ = 0.9914	*R* ^2^ = 0.9997
BG6 (SIF)	*K* = 25.06	*K* = 0.370	*K* = 0.0186	*K* = 0.485 *n* = 1.0000
	*R* ^2^ = 0.9988	*R* ^2^ = 0.9861	*R* ^2^ = 0.9831	*R* ^2^ = 0.9995
BG7 (SGF)	*K* = 20.036	*K* = 0.269	*K* = 0.0234	*K* = 0.496 *n* = 1.0000
	*R* ^2^ = 0.9999	*R* ^2^ = 0.997	*R* ^2^ = 0.9916	*R* ^2^ = 0.9998
BG7 (SIF)	*K* = 23.812	*K* = 0.346	*K* = 0.0197	*K* = 0.502 *n* = 1.0000
	*R* ^2^ = 0.9998	*R* ^2^ = 0.9958	*R* ^2^ = 0.9928	*R* ^2^ = 0.9997

The fitting results indicated that zero‐order and Korsmeyer–Peppas models showed high fitting accuracy for most formulations, suggesting that FA release was not governed by a single transport mechanism. The *n* values obtained from the Korsmeyer–Peppas model were close to unity, indicating that polymer relaxation and structural rearrangement of the BG matrix contributed significantly to FA release, together with diffusion processes. Therefore, the release behavior was considered to result from the combined effects of matrix organization, molecular mobility, and structural relaxation.

In BGs, higher BW content might have restricted gastric fluid penetration into the gel matrix, leading to slower FA release. This behavior could also be associated with the lower swelling ratio, and the hydrophobic nature of BW, which created a more complex pathway for fluid penetration and FA transport, thereby contributing to a more controlled release profile (Zheng et al. 2020). Similarly, Mazurkevičiūtė et al. (2022) reported that reducing viscosity in BG formulation resulted in faster release of encapsulated bioactive compounds (Mazurkevičiūtė et al. 2022).

In samples containing 7% BW, a higher proportion of Na‐Cas appeared to correlate with reduced FA release during the gastric phase. This behavior may be related to the low pH of the gastric environment, where Na‐Cas approaches its isoelectric point, decreasing solubility and promoting aggregation. Such aggregation may decrease matrix relaxation and limit structural rearrangement, thereby slowing FA transport through the BG network (Akhtar et al. 2022). Conversely, increasing CS content in the BG matrix enhanced FA release during the gastric phase, which can be attributed to the pH‐dependent behavior of CS. Under acidic conditions, protonation of amino groups increases polymer hydration and chain mobility, which may facilitate matrix relaxation and promote FA transport through the network.

In addition to matrix composition, molecular interactions between FA and the BG components may also play a key role in controlling the release behavior. Hydrogen bonding between FA hydroxyl groups and functional groups of CS or Na‐Cas, along with hydrophobic partitioning of FA within the OG phase, can enhance FA retention within the dual‐network structure. These interactions may improve FA stability and reduce premature FA migration within the dual‐network structure, particularly under acidic gastric conditions. (Do et al. 2022).

This trend was significantly reversed during the SIF phase, where higher Na‐Cas content resulted in increased FA release. This behavior may be attributed to the improved hydration and solubilization of Na‐Cas under intestinal conditions, together with its susceptibility to enzymatic degradation by proteases. These changes can promote matrix relaxation and structural rearrangement, facilitating FA transport through the BG network (Salehi et al. 2023). Similar findings were reported by Eghbal et al. (2019), where higher Na‐Cas content increased solubility and accelerated release and migration of natamycin incorporated in them (Eghbal et al. 2019).

In another study conducted by Wang et al. (2023), designed nanocomposites containing Na‐Cas and carboxymethylpachymaran for curcumin release demonstrated a highly controlled release profile (Wang et al. 2023). In contrast, higher CS content reduced FA release during the intestinal phases. This behavior may be related to the reduced protonation of CS amino groups under alkaline conditions, which decreases its hydration capacity and may increase hydrophobic interactions within the polymer network. Consequently, the reduced polymer solubility and mobility may limit FA migration. In addition, the resistance of CS to digestive enzymes duo to the stability of its β‐(1 → 4)‐glycosidic bonds may contribute to the sustained retention of FA within the BG matrix (Perez‐Salas et al. 2025; Yang, Zheng, et al. 2024).

After two hours of each digestion phase, approximately 60%–90% of the FA was released across all samples. The highest release rate, around 90%, was observed for BG7, which could be attributed to its lower BW content and more porous structure, resulting in enhanced fluid absorption and reduced hydrophobicity. The differences observed among formulations were consistent with the kinetic modeling results, which indicated that variations in BG composition altered the structural relaxation and transport behavior of FA within the matrix for BG7, likely due to its low BW content and more porous matrix, which increased fluid absorption and reduced hydrophobicity (Ebrahimzadeh and Koocheki 2026). In contrast, BG4 containing the highest CS content, exhibited the lowest release rate (approximately 60%). This behavior may be related to the formation of a denser polymer network, which restricted FA mobility and delayed its transport. The kinetic analysis further supported that variations in BG composition influenced the structural relaxation and transport behavior of FA within the matrix. These findings indicate that adjusting BW content and the Na‐Cas ratio can effectively modulate FA release behavior and achieve a more controlled release profile. Although a stronger gel network may restrict early fluid penetration and delay FA release during gastric digestion, changes in intestinal conditions and enzymatic activity may alter the release behavior during the intestinal phase (Liu et al. 2025). Walendziak et al. (2025) also reported that the optimized biopolymer combinations in BGs can achieve targeted and sustained delivery of bioactive compounds through different digestion phases. (Walendziak et al. 2025). This release pattern highlighted the importance of fine‐tuning BW content and CS: Na‐Cas for phase specific FA delivery, which is especially advantageous in nutraceutical formulations requiring stability in gastric conditions and efficient intestinal release.

Overall, FA release behavior reflected the combined effects of formulation composition, intermolecular interactions, microstructure, and viscoelastic properties (Sinha et al. 2024). The kinetic modeling results, together with SEM, FTIR, swelling, and rheological analyses, demonstrated that FA release was governed by the combined influence of matrix organization, polymer relaxation, and molecular transport within the BG structure. Formulations exhibiting stronger gel networks, higher viscosity, and denser microstructures generally showed lower swelling and slower FA transport, whereas weaker and more heterogeneous structures facilitated fluid penetration and enhanced FA release. These findings indicate that the controlled release performance of the developed BGs resulted from the synergistic contribution of HG composition, OG reinforcement, and the structural organization of the dual‐network system (Sinha et al. 2024; Karimidastjerd et al. 2024).

Although swelling behavior is an important factor affecting FA transport, the release profile of BGs was not solely governed by water uptake (Zhang et al. 2025; Valizade et al. 2026). The nonlinear relationship between swelling and FA release may result from the combined effects of network strength, hydrophobic barriers created by BW, intermolecular interactions between FA and polymer components, and microstructural heterogeneity (Zhang et al. 2025; Xie et al. 2023; Valizade et al. 2026). This interpretation is further supported by the kinetic analysis, indicating that matrix relaxation and structural organization also contributed to the release mechanism. Therefore, formulations with higher swelling do not necessarily exhibit proportionally higher release rates, as the internal organization and resistance of the gel network also play critical roles in controlling FA transport (Zhang et al. 2025).

Compared with CS‐only hydrogels, incorporation of the oleogel phase into the BG structure introduced hydrophobic domains that reduced rapid water penetration and delayed premature FA release during the gastric phase (Valizade et al. 2026; Zhang et al. 2025). Conversely, compared with BW‐only oleogels, the continuous HG network improved the structural integrity of the system and promoted a more controlled release under intestinal conditions (Valizade et al. 2026). Therefore, the dual‐network architecture combined the complementary functions of both phases, resulting in a more balanced release profile than that typically observed in single‐phase delivery systems. Overall, the independent kinetic modeling of the gastric and intestinal phases, together with SEM, FTIR, swelling, and rheological analyses, demonstrated that FA release from the developed BGs was governed by the combined effects of matrix organization, polymer relaxation, intermolecular interactions, and molecular transport. Additionally, encapsulation efficiency was not evaluated in the present study, and future investigations should combine EE% analysis with release characterization to better understand FA loading capacity and retention mechanisms within the BG matrix.

### 3.7 Comparison With Previously Reported Food‐Grade Bigel Systems

Compared with previously reported food‐grade BG systems, the formulations developed in this study exhibited elastic‐dominant behavior (*G*′ > *G*″), controlled swelling, and sustained FA release which can be attributed to the synergistic combination of a polysaccharide–protein HG and a BW‐based OG (Valizade et al. 2026; Sinha et al. 2024). Similar to previous studies, increasing BW concentration reduced swelling and enhanced the structural compactness of the gel network, whereas increasing Na‐Cas content promoted water uptake and facilitated FA release under intestinal conditions (Karimi et al. 2026; Xie et al. 2023). Compared with single‐phase HGs or OGs, the present dual‐network structure provided a balanced combination of mechanical stability and controlled release behavior under simulated gastrointestinal conditions (Sinha et al. 2024; Xie et al. 2023). However, despite the favorable physicochemical and release properties, additional studies on storage stability, thermal processing tolerance, and sensory attributes are still required before practical food applications can be fully considered.

## 4 Conclusion

Novel BG formulations were developed based on L and BW OG and CS and Na‐Cas, HG as carriers for the controlled release of FA. The results demonstrated that BW concentration and the CS: Na‐Cas ratio appeared to have significant effects on the structural, rheological, and release properties of the BGs. Higher BW concentrations seemed to result in more compact and stable gel networks, which reduced swelling and slowed FA release. Similarly, higher CS content appeared to enhance stability and delay release in intestinal conditions due to its reduced solubility at neutral pH. In contrast, higher Na‐Cas levels tended to promote faster release, especially in the intestinal phase, likely due to increased solubility and enzymatic degradation. These observations indicate that fine‐tuning the HG and OG composition could modulate release profiles, suggesting potential applications in food and nutraceutical delivery systems where the protection and targeted delivery of bioactive compounds are essential. Among the developed formulations, BG4 exhibited superior structural stability, as evidenced by its higher viscoelastic properties, lower swelling ratio, and compact microstructure observed in SEM analysis. In contrast, BG5 showed the most favorable release profile by minimizing FA release under simulated gastric conditions while prompting greater release during intestinal phase, indicating a controlled and targeted release.

This study highlights the potential of BGs for controlled release of FA, with a dual‐network structure formed by CS/Na‐Cas HG and L/BW OG. By optimizing the HG: OG composition, both stability and release can be precisely tuned for functional food applications. Therefore, the selection of the most suitable BG formulation depends on the intended application; BG4 may be advantageous for applications requiring enhanced structural stability, whereas BG5 represents a promising candidate for targeted intestinal delivery of FA due to its desirable release behavior. However, this study had some limitations. Encapsulation efficiency (EE%) of FA was not determined in the present study, which represents a limitation that should be addressed in future investigations to provide further insight into the loading capacity and retention efficiency of the developed BG systems. Regulatory considerations, cost efficiency, process scalability, and performance under industrial processing conditions were not explored in this study. Future investigations are therefore required to validate the system under real conditions and assess its commercial feasibility. Furthermore, although the developed BG system exhibited favorable physicochemical and release characteristics compared with previously reported food‐grade BG systems, further investigations on storage stability, thermal processing tolerance, and sensory attributes are required before practical industrial applications.

From a practical perspective, the use of food‐grade and widely available ingredients such as BW, L, CS, and Na‐Cas suggests a favorable potential for large‐scale production. Further studies should evaluate cost efficiency, process scalability, regulatory compliance, and performance under industrial processing conditions to support future implementation in food and nutraceutical applications. From a functional food perspective, the BG system developed in this study provides a food‐grade strategy for protecting FA during gastric conditions while enabling controlled release in the intestinal phase. The dual‐network structure, formed by a CS/Na‐Cas HG and a BW‐based OG, allows modulation of release behavior through compositional tuning rather than pharmaceutical excipients. This approach highlights the potential of BGs for functional foods and nutraceutical applications requiring stable and targeted bioactive delivery.

## Author Contributions

**Ghazal Kalantari:** supervision, conceptualization, writing – review and editing, investigation. **Fatemeh Valizadeh:** methodology, software. **Khadije Abdolmaleki:** supervision, conceptualization, writing – review and editing, investigation.

## Funding

This work was supported by the Kermanshah University of Medical Sciences, 4020660.

## Conflicts of Interest

The authors declare no conflicts of interest.

## Data Availability

The data that support the findings of this study are available from the corresponding author upon reasonable request.
